# Submucosal mucoid as a late complication after appendectomy—A case report

**DOI:** 10.1016/j.ijscr.2019.03.051

**Published:** 2019-04-05

**Authors:** Kristýna Bajcurová, Petr Novák, Eva Korčáková, Hynek Mírka, Jan Geiger, Petr Rajal, Ondřej Daum, Marcela Podolcová

**Affiliations:** a Department of Imaging Methods, Charles University Medical School and Teaching Hospital in Pilsen, Alej Svobody 923/80, 304 60, Pilsen, Czech Republic; b Biomedical Centre, Charles University Medical School in Pilsen, Alej Svobody 1655/76, 323 00, Pilsen, Czech Republic; c Department of Surgery, Charles University Medical School and Teaching Hospital in Pilsen, Alej Svobody 923/80, 304 60, Pilsen, Czech Republic; d Department of Internal Medicine, Klatovy Hospital, Plzeňská 929, 339 01, Klatovy II, Czech Republic; e Pathological-anatomical Šikl Institute, Charles University Medical School in Pilsen, Teaching Hospital in Pilsen, Dr. E. Beneše 13, 305 99, Pilsen, Czech Republic; f Radiological Department, Klatovy Hospital, Plzeňská 929, 339 01, Klatovy II, Czech Republic

## Abstract

**Introduction:**

Shortly after an operation infection, intraabdominal abscess, seroma, haemorrhage or development of paralytic ileus may occur. Postoperative adhesions, nonspecific abdominal pain without signs of obstruction, incisional hernia and appendicitis or mucocele in appendiceal stump present common late complications. We present a unique case of a late complication after appendectomy.

**Presentation of Case:**

The case report describes a unique expansion in the ascending colon of a young athlete with long-lasting abdominal pain in the lower right quadrant. Colonoscopy showed a lesion in a wall of the ascending colon. Computed tomography (CT) confirmed a cystoid formation of high content density in a wall of the caecum. A right hemicolectomy was performed. Histology showed a lesion located in the submucosa with intestinal lining and stroma rich in lymphoid cells. These are the typical attributes of the wall of the appendix. Other parts of the wall were not demonstrated, and there was no communication with the lumen of the native bowel.

**Conclusion:**

A submucosal cavity filled with acellular matter, which were probably disintegrated epithelioid structures, and calcifications were found in the ascending colon, while no intestinal cell atypia or dysplasia was found. The case cannot be classified under any previously presented diagnosis.

## Highlights

• We present a unique case of a late complication after appendectomy.
• Histology showed attributes of the appendix wall, the muscularis propria was missing.
• A rare complication after appendectomy, according to our knowledge not published yet.
• A possible descriptive term might be a submucosal mucoid.

## 1 Introduction

Appendectomy is one of the most common surgical operations. Shortly after operation, complications occur in 11–16 % of cases and include wound infection, intraabdominal abscess, seroma, bleeding or development of paralytic ileus [1]. Postoperative adhesions, nonspecific abdominal pain without signs of obstruction, and incisional hernia represent common late complications [2]. Residual appendiceal tissue remaining after operation may cause the development of stump appendicitis, presented by the same symptoms as appendicitis, and higher risk of perforation [3]. A partially removed appendix leaves a stump, where the abnormal accumulation of mucus and dilatation of the lumen leads to the development of mucocele, mostly secondary to neoplasia [[4], [5], [6], [7], [8]].

The case report describes a unique complication, which is previously absent from the literature.

Our work has been reported in line with the SCARE criteria. [9]

## 2 Presentation of case

A 23-year-old male, with a history of appendicitis 9 years ago and a previous operation for an inguinal hernia was admitted to our teaching hospital due to long-term intermittent abdominal pain in the right lower quadrant. The pain was aggravated by intensive training. Discomfort graduated to changes in bowel habit, such as constipation, flatulence, dyspepsia with nausea and impaired digestion. Over the previous 6-month period, a weight loss of 5 kg occurred.

**Fig. 1:**
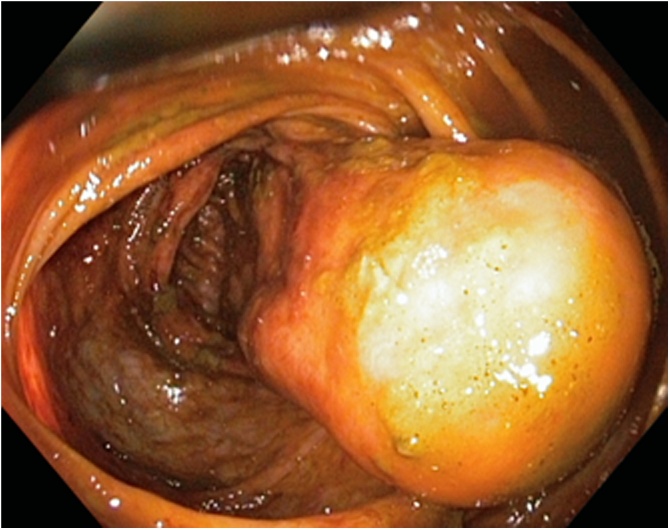
Colonoscopy finding. Colonoscopy revealed a submucosal cylindrical formation in the wall of the ascending colon and protruding into the lumen.

A physical examination showed only a smooth scar after appendectomy, with tenderness in the right lower abdominal quadrant during deep palpation but without palpable resistance. A colonoscopy, performed at the district hospital, revealed a submucosal cylindrical formation in the wall of the ascending colon protruding into the lumen (Fig. 1). There were two attempts to perform a biopsy at the district hospital, but without a valid result. After an admission to our teaching hospital there were no other attempts performed, because it would cause another delay in the performance of the operation.

**Fig. 2:**
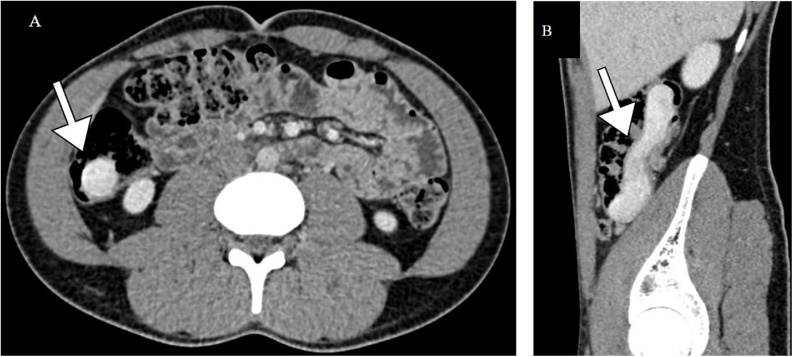
CT examination. A CT examination showed an encapsulated formation with high attenuation in the inner layer of the bowel wall. The lesion is marked by an arrow in axial image (A) and sagittal image (B).

A CT scan was performed in arterial, venous and late phase after the administration of iodinated contrast medium in the peripheral vein (Fig. 2). The scan revealed a well - encapsulated formation of high attenuation with a density of approximately 160 HU (Hounsfield units). The formation was homogenous and almost identical in all performed phases, and there was no significant change in contrast enhancement. The formation was located in the wall of the caecum and ascending colon, and it was protruding into the lumen. There were no pathological changes in the surrounding area, and no obturation of the lumen. Multiple enlarged lymph nodes were found in the mesentery. There were normal findings for other abdominal organs.

**Fig. 3:**
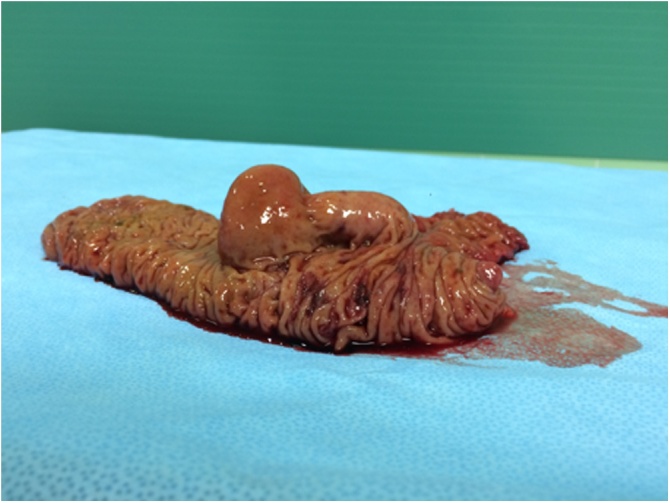
Operation finding. After a right hemicolectomy, a lesion located in the intestinal submucosa and protruding into the lumen was confirmed.

Based on a multidisciplinary consensus, the patient was indicated for a laparoscopically assisted right hemicolectomy under general anaesthesia. A surgical team, specialized in laparoscopic abdominal operation, performed the hemicolectomy 3 months after the expansion was diagnosed. During the operation, there were no pathological findings for other abdominal organs. There were adhesions to the scar after appendectomy, which were disrupted. The formation in the ascending colon was not found after mobilization of the colon from the parietal peritoneum; therefore, a perioperative colonoscopy was performed. The colonoscopy confirmed that the formation in the caecum extended to the ascending colon. After the examination, the laparoscopically assisted right hemicolectomy and mesenteric lymphadenectomy were performed. Approximately 13.5 cm of colon without appendix and 6 cm of terminal ileum were removed (Fig. 3). During the inspection of the removed colon, a cylindrical submucosal lesion, containing mushy mucous matter and measuring 7.5 × 5.5 × 2 cm, was found. There were normal findings in the rest of the removed ileum, colon and 25 lymph nodes.

**Fig. 4:**
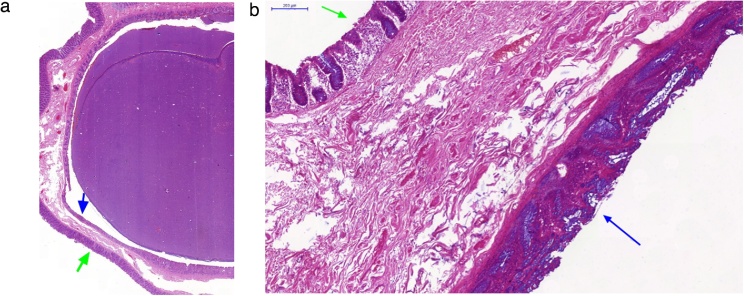
Histology. (A) Original magnification x40, haematoxylin and eosin stain. Photomicrograph showed submucosal localization of the cavity lined by the intestinal mucosa. Cavity and native bowel lumen were separated by only a thin layer of submucosal matter. (B) Original magnification x200, haematoxylin and eosin stain. The photomicrograph confirms the absence of the muscularis propria between the inner layer of the cavity (black arrow) and the inner layer of the caecum (white arrow). There was a disruption of the crypt architecture in the inner layer of the cavity with stroma rich in lymphoid tissue.

Histologically, there was a submucosal cavity lined by intestinal mucosa with stroma rich in lymphoid tissue, while no other layer of the wall was found. There was no communication with native bowel lumen (Fig. 4). The cavity was filled with a large amount of acellular structures, which were most likely disintegrated epithelium structures, and calcifications, with only a small amount of mucous, and there were no intestinal contents.

Shortly after operation a laboratory examination showed an elevation of the white cell count to 21 × 10^9^/L and a CRP (C-reactive protein) level up to 207 mg/L, probably due to the reaction to an operation and systemic inflammatory reaction, the next day a laboratory examination showed no disturbances. In few days the patient made an uneventful recovery and was discharged home within a week. Three weeks after operation, the patient was without any difficulties. Patient was seen regularly for 3 months, without any complication, he was forwarded to follow-up at a district hospital.

## 3 Discussion

Considering the history, age, location of the lesion, clinical condition, CT findings and after correlation with the macroscopic and histological findings, the residual epithelium of the appendix with cystic transformation appeared as the most likely diagnosis. Appendiceal tissue was probably dislocated to the submucosa of the caecum during the appendectomy. The cavity was forming for 9 years, filling with disintegrating epithelial structures, and over the years it grew to a size that caused clinically significant difficulties. Small calcifications are typical of the disintegrating epithelial cells. In the available literature, similar complications of appendectomy have not yet been described. In differential diagnosis, the cystic lesions, congenital abnormalities, tumours and postoperative complications were considered. Particularly, we tend to exclude mostly duplications, congenital or acquired diverticulum and appendiceal mucocele.

Gastrointestinal duplications are rare congenital anomalies located anywhere within the gastrointestinal tract. The most common location is the terminal ileum.

There are three criteria required for diagnosis of duplication, which are as follows:

first, a connection to the gastrointestinal tract, which commonly means either the serous or muscle wall layer;

second, an alimentary tract-type mucosal epithelial lining;

third, contains all 3 layers of bowel wall, including the muscularis propria.

Imaging methods, such as CT, ultrasonography (USG) or magnetic resonance (MR), are a very important part of the diagnostic process. In CT examination, spherical or tubular duplication cysts are distinguished, they do not infiltrate adjacent structures or organs, and the wall might be denser, but the content density reaches 0–20 HU, with no significant contrast enhancement. A rare case appeared in the literature [10], wherein the content reached a density of 40 HU. In USG, cyst-like lesions with homogenous amorphous content can be seen, usually with an inner hyperechoic layer and outer hypoechoic muscle layer. MR examination shows a low signal in the T1-weighted images and a high signal in the T2-weighted images.

Duplications are a rare diagnosis. The duplications, although benign, have the potential to become malignant in up to 23% of cases; therefore, resection is recommended as a method of treatment [11].

The presence of the muscularis propria in the wall and a communication with the alimentary tract are the attributes of either congenital or acquired diverticulum. The muscularis propria was not proven in the presented case, and therefore the diagnosis of diverticulum was excluded. Communication with the alimentary tract was not proven either.

Appendiceal mucocele is a rare acquired condition found in the stump after less than 1% of appendectomies [12]. We were able to find only 9 references to mucocele in the appendiceal stump [[4], [5], [6], [7], [8],^13^,^14^], but none demonstrated submucosal location. Mucocele is defined as a dilated appendiceal lumen resulting from the abnormal accumulation of mucus. There are 4 histological subtypes: retention cyst, mucosal hyperplasia, low-grade appendiceal mucinous neoplasm and mucinous adenocarcinoma. Neither degenerative changes typical of retention cysts, nor neoplastic changes in the mucosa lining cells were found. Mucocele often occurs asymptomatically; in 23–50 % of cases, it is an incidental finding during USG, CT examination or during surgery for different reasons. In other cases, in which the patient does not have specific difficulties, it usually starts as right lower abdominal quadrant pain, but obstruction and change in bowel habits or bleeding into the gastrointestinal tract may appear [15,16]. USG findings are variable, but cystic lesions with anechoic fluid or hypoechoic masses are usually found [17]. It is found a low-density content of approximately 0–20 HU in CT images and, according to the amount of water and fat in the lesion, we describe a low signal in the T1-weightet image and high signal in the T2-weighted image during MR examination. Infection and rupture with the development of pseudomyxoma peritonei are dangerous complications of non-neoplastic mucocele [12]. In these cases, surgery is generally recommended as a method of treatment, and biopsy is usually not performed.

In the case of our patient, the high-density content was caused by a large number of calcifications, which usually evolve during long-term congestion. The cavity was located intimately to the stump after appendectomy, and it reached the hepatic flexure as it grew. Since the muscularis propria was missing, we conclude that the original appendiceal mucosa cells transformed cystically after being dislocated to the submucosa of the caecum during surgery.

## 4 Conclusion

The described late complication after appendectomy is rare and according to our knowledge it has not yet been published. A possible descriptive term might be a submucosal mucoid.

## Conflicts of interest

We declare that we have no competing interests.

## Sources of funding

This study was supported by the National Sustainability Program I (NPU I) Nr. LO1503 provided by the Ministry of Education Youth and Sports of the Czech Republic and grant SVV 260392 provided by Charles University and by the project of the Ministry of Health of the Czech Republic - Conceptual Development of Research Institutions00669806 – FN Plzen, by the project of the Charles University Prague Progress Q39, by the project Centrum of clinical and experimental liver surgery “UNCE/MED/006 of the Charles University and by the project CZ.1.05/2.1.00/03.0076 from European Regional Development Fund.

The role of the donators was funding the grammar proof-reading by American Journal Experts and the publication related costs.

## Ethical approval

Ethical approval has been exempted by our institution. No identifiable patient’s parts were seen and all of the data were collected from clinical records and imaging systems for routine perioperative planning.

## Consent

Written informed consent was obtained from the patient for publication of this case report and accompanying images. A copy of the written consent is available for review by the Editor-in-Chief of this journal on request.

## Author contribution

**Kristýna Bajcurová**: Writing – Original Draft, Resources, Visualization. **Petr Novák**: Resources, Writing – Review and Editing. **Jan Geiger**: Resources. **Eva Korčáková**: Resources, Writing – Review and Editing, Supervision. **Hynek Mírka** – Writing – Review and Editing, Supervision. **Petr Rajal**: Resources. **Marcela Podolcová** – Resources. **Ondřej Daum**: Resources, Writing – Review and Editing.

All authors read and approved the final manuscript.

## Registration of research studies

Not applicable.

## Guarantor

Kristyna Bajcurova

## Provenance and peer review

Not commissioned, externally peer-reviewed.
